# Roles of the Cell Surface Architecture of *Bacteroides* and *Bifidobacterium* in the Gut Colonization

**DOI:** 10.3389/fmicb.2021.754819

**Published:** 2021-10-14

**Authors:** Keita Nishiyama, Tatsunari Yokoi, Makoto Sugiyama, Ro Osawa, Takao Mukai, Nobuhiko Okada

**Affiliations:** ^1^Department of Microbiology and Immunology, Keio University School of Medicine, Tokyo, Japan; ^2^Department of Microbiology, School of Pharmacy, Kitasato University, Tokyo, Japan; ^3^Laboratory of Veterinary Anatomy, School of Veterinary Medicine, Kitasato University, Towada, Japan; ^4^Research Center for Food Safety and Security, Kobe University, Kobe, Japan; ^5^Department of Animal Science, School of Veterinary Medicine, Kitasato University, Towada, Japan

**Keywords:** adhesion, *Bifidobacterium*, *Bacteroides*, mucin, mucosal bacteria, cell surface protein, colonization

## Abstract

There are numerous bacteria reside within the mammalian gastrointestinal tract. Among the intestinal bacteria, *Akkermansia*, *Bacteroides*, *Bifidobacterium*, and *Ruminococcus* closely interact with the intestinal mucus layer and are, therefore, known as mucosal bacteria. Mucosal bacteria use host or dietary glycans for colonization *via* adhesion, allowing access to the carbon source that the host’s nutrients provide. Cell wall or membrane proteins, polysaccharides, and extracellular vesicles facilitate these mucosal bacteria-host interactions. Recent studies revealed that the physiological properties of *Bacteroides* and *Bifidobacterium* significantly change in the presence of co-existing symbiotic bacteria or markedly differ with the spatial distribution in the mucosal niche. These recently discovered strategic colonization processes are important for understanding the survival of bacteria in the gut. In this review, first, we introduce the experimental models used to study host-bacteria interactions, and then, we highlight the latest discoveries on the colonization properties of mucosal bacteria, focusing on the roles of the cell surface architecture regarding *Bacteroides* and *Bifidobacterium*.

## Introduction

A diversity of microorganisms co-exists with humans; the estimated total number of bacteria in the human body (for a reference weight of 70kg) is approximately 3.8×10^13^ (Sender et al., 2016a,b). Large-scale sequencing analyses, including those in the Human Microbiome Project and Metagenomics of the Human Intestinal Tract (Meta-HIT) study, revealed some common patterns of the composition of the human microbiome (Qin et al., 2010; Human Microbiome Project Consortium, 2012). The human gut is composed of hundreds of bacterial taxa (at the species level), typically dominated by five major phyla; Firmicutes, Bacteroidetes, and Proteobacteria that are the most abundant, with Actinobacteria and Verrucomicrobia as relatively minor components. Interestingly, the adult intestinal microbiota is partially stable, with a core of 33–40 bacterial species (accounting for 75% of the abundance of gut microbiota) persisting for at least 1year in individuals (Martínez et al., 2013). In the human colon, the density of bacteria – mainly anaerobic bacteria of the families Bacteroidaceae, Prevotellaceae, Rikenellaceae, Lachnospiraceae, and Ruminococcaceae – reaches 10^11^/g (Donaldson et al., 2015).

**Figure 1:**
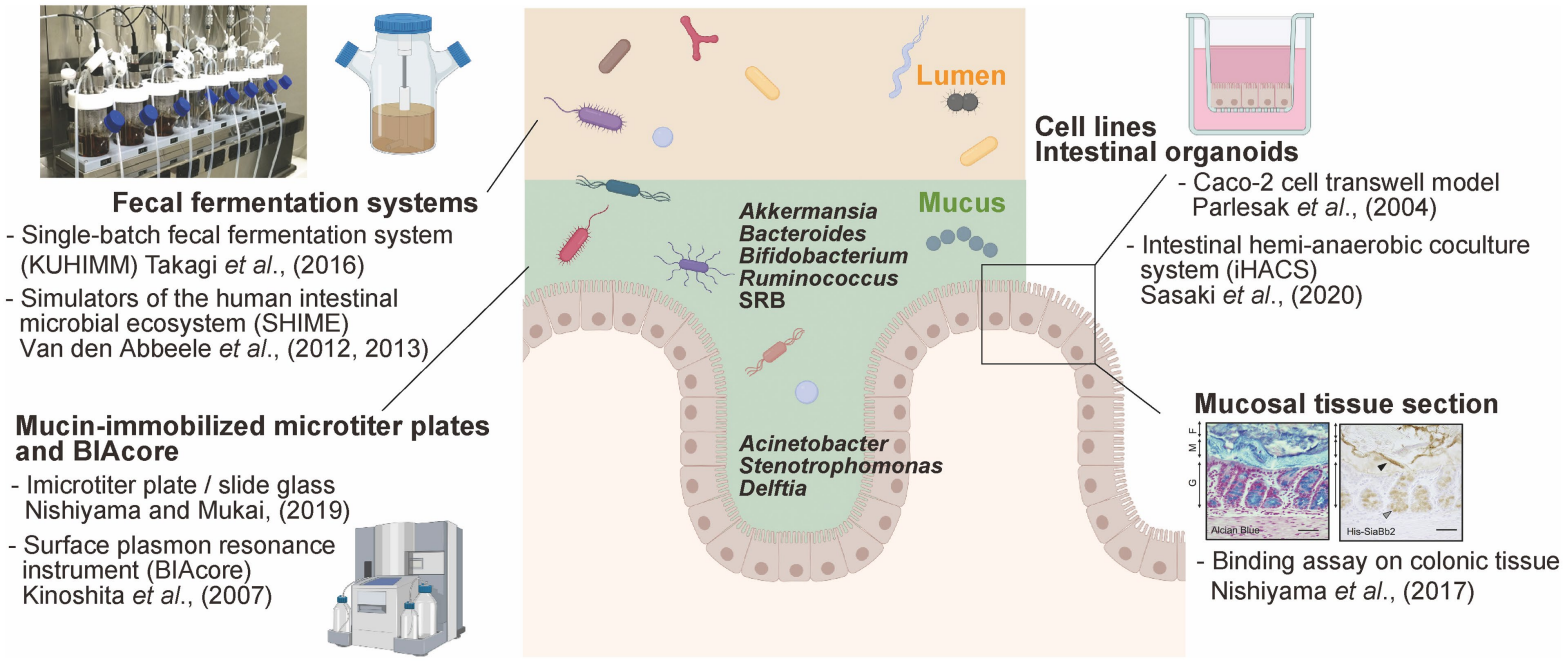
Available experimental models to study mucus-bacteria interactions. Schematic summary of experimental models of interactions between gut bacteria and mucus secreted by enterocytes. This figure was adapted from Takagi et al. (2016) and Nishiyama et al. (2017) and created using BioRender (https://app.biorender.com).

Mucin glycoproteins are secreted from goblet cells form two distinct mucus structures in the mammalian colon: a gel-like outer mucus layer and an inner mucus layer (Johansson et al., 2008). Mucins are heavily *O*-glycosylated and can also be *N*-glycosylated, albeit much more sparsely. The mucus layers protect the epithelia, as well as the respiratory and urinary tract, against pathogens and mechanical damage. The gel-like outer mucus layer is associated with a unique microbial community comparted with the planktonic lumen microbiome (Nava et al., 2012; Li et al., 2015). For example, several studies indicated that the outer mucus layer is enriched in mucin-degrading/consuming bacteria, such as *Akkermansia muciniphila* (in mice and humans; Png et al., 2010; Derrien et al., 2011), *Bacteroides fragilis* (in mice; Huang et al., 2011; Lee et al., 2013), *Bacteroides thetaiotaomicron* (in mice; Eckstein et al., 2020), *Bacteroides vulgatus* (in humans; Png et al., 2010), *Ruminococcus gnavus* (in humans; Png et al., 2010), *Ruminococcus torques* (in humans; Png et al., 2010), and *Bifidobacterium bifidum* (in humans; Ruas-Madiedo et al., 2008; Png et al., 2010; Figure 1). The human mucus layer is also persistently colonized by hydrogenotrophic microbes, including sulfate-reducing bacteria (SRB), such as *Desulfovibrio piger*, *Desulfovibrio desulfuricans*, and *Bilophila wadsworthia* (Nava et al., 2012). Some of the bacterial species present in the mucus show different proliferation and resource utilization abilities depending on the niche (mucus versus intestinal lumen), according to their genome-encoded metabolic repertoire (Jakobsson et al., 2015; Li et al., 2015; Donaldson et al., 2020). Therefore, the specific composition of mucus-associated microbiome potentially affects the intestinal mucus physiological barrier, with foreseeable implications for the host health and disease (Jakobsson et al., 2015).

In contrast, the tightly adhering inner mucus layer and crypts are penetrated at low density by a more restricted community. Histological analysis using Warthin-Starry staining and 16S rRNA fluorescence *in situ* hybridization allowed the detection of the genera *Acinetobacter*, *Stenotrophomonas*, and *Delftia* (phylum Proteobacteria) within murine intestinal crypts (Stedman et al., 2019; Figure 1). These bacteria are hardly detected in the colonic luminal contents because they require oxygen for respiration; importantly, oxygen is supplied from epithelial cells, enabling the growth of these bacteria within the crypts and the maintenance of a specific microbiome. It has been shown that the lipopolysaccharide of *Acinetobacter* acts as a physical stimulator of intestinal stem cells, located close to the bottom of the crypt, impacting their differentiation (Naito et al., 2017). Thus, the distinct spatial distribution of bacteria in the intestinal crypts (within the mucus axis) is probably due to strategic bacterial colonization processes depending on complex interactions with the host.

This review provides an overview of *in vitro* experimental models that demonstrate and visualize host-bacteria interactions. We then evaluate recent insights into the colonization properties of mucosal bacteria and focus on the roles of the cell surface architecture in *Bacteroides* and *Bifidobacterium*. For example, *Bacteroides* regulates the cell surface architecture to effectively colonize the intestinal tract; such alterations enable the spatial distribution of *Bacteroides* in the mucus-epithelium (Donaldson et al., 2020). Moreover, co-existence of intestinal bacteria can stimulate the production of extracellular vesicles (EVs), thereby providing adhesive advantages for *Bifidobacterium* (Nishiyama et al., 2020). Elucidation of the colonization strategies of mucosal bacteria may help clarify the mechanisms by which these microorganisms survive in the gut.

## Experimental Models to Study Host-Bacteria Interactions

Information regarding the mechanisms that underlie interactions between bacteria and the host is important to understand the effects of the microbiota on gut homeostasis, including microbiota-induced immunomodulation. Germ-free mouse models are generally considered the gold standard for microbiome research, especially to explore the close links between bacteria and the host (Russell et al., 1965). For instance, segmented filamentous bacteria (SFB) belonging to the Firmicutes phylum can resist *in vitro* culturing (Schnupf et al., 2015); however, the authors reportedly stably colonize the gut of germ-free mice (in the context of a mono-association system) probably due to the tight adhesion to the small intestinal epithelium (Ivanov et al., 2009). This study also showed that SFB also impact intestinal immune function and phenotype *in vivo* (Ivanov et al., 2009). To analyze the role and function of each bacterial species on the distribution of other species, as described in the previous section, several kinds of experimental *in vitro* models mimicking the mucus, intestinal epithelium, and planktonic lumen have been developed (Figure 1). Importantly, *in vitro* models offer a major advantage; external factors can be eliminated as much as possible, allowing a better mechanistic clarification of the microbe-host interactions.

### Mucin-Immobilized Microtiter Plates and BIAcore

Mucin from the gastrointestinal (GI) tract immobilized into microtiter plates has been used for *in vitro* bacterial adhesion assay, particularly with *Lactobacillus* and *Bifidobacterium* (for a detailed protocol, please refer to Nishiyama and Mukai, 2019). Commercial porcine gastric mucin has been commonly used; however, colonic mucin isolated and purified from human intestinal biopsy or porcine colon tissues using gel filtration chromatography has also been used for adhesion tests (Uchida et al., 2004; Kinoshita et al., 2007; Nishiyama et al., 2014). Mucin is immobilized into microtiter plates and exposed to bacterial suspension. Adhered bacteria are then determined (after the required washing steps) *via* the direct quantification of viable cells, or using quantitative PCR. Alternatively, adhesion can be quantified using crystal violet staining (Collins et al., 2012) or fluorescent dye staining (Mackenzie et al., 2010; Etzold et al., 2014). Using this experimental approach, *B. fragilis*, capable of adhering to the murine mucus layer *in vivo*, adhered well to both murine and porcine colonic mucin-immobilized microtiter plates (Huang et al., 2011). These data suggest that the adhesion phenotype determined using this *in vitro* experimental approach can be translated to the *in vivo* context.

However, the above-described method is not suitable for the study of some bacterial species. For instance, *A. muciniphila* (one of the mucus-associated bacterial species) adhered to human colonic cell lines and extracellular matrix (ECM) proteins but not to human colonic mucin-coated microtiter plates *in vitro* (Reunanen et al., 2015). The authors proposed that *A. muciniphila* might have detached from the immobilized mucus due to their mucin-degrading enzymatic activity. In addition, it is noticed that the purity of mucin directly affects bacterial adhesion. The adhesion capacity of several *Bifidobacterium* remarkably differed in the context of density-gradient ultracentrifugation-derived high purity mucin versus single gel filtration chromatography-derived crude mucin (Nishiyama et al., 2014).

Alternatively, the surface plasmon resonance instrument BIAcore can be used to measure the adhesion of bacteria to mucin and glycol-conjugates (Uchida et al., 2004; Kinoshita et al., 2007). Purified mucin can be immobilized on the BIAcore sensor chip *via* an amine coupling reaction, while bacteria are injected as the analyte. The amount of adhered bacterial is determined based on resonance units (1 RU=1pg/mm^2^). The advantage of BIAcore-based analyses is the ability to trace ligand-analyte interactions over time. This experimental approach was also used to screen bacteria with the ability to adhere to mucin sulfo- and sialyl-sugar chains using mucin pre-treated with sialidase and sulfatase (Huang et al., 2013; Nishiyama et al., 2014).

### Mucosal Tissue Sections

The intestinal region-specific adhesion ability of bacteria, such as pathogenic *Escherichia coli* O78 (Edelman et al., 2003) and different *Lactobacillus* strains (Edelman et al., 2002), was investigated using frozen sections of the alimentary tract of chicken, as well as mucus from the ileum. However, while this method is suitable for the visualization of the adhesion properties of bacterial cells, it does not allow the objective quantification of bacterial adhesion. On the other hand, bacterial adhesion factors can be expressed in *E. coli* as recombinant proteins and used for histological staining. Methacarn fixative and paraffin embedding are one of the most effective methods for preserving mucus integrity (Nishiyama et al., 2016a). For example, the pilus protein from *Lactobacillus rhamnosus* GG (Nishiyama et al., 2016b), a sialidase from *B. bifidum* ATCC15696 (Nishiyama et al., 2017), and the sialic acid-binding carbohydrate-binding module (CBM40) conserved in *trans*-sialidase (RgNanH) from *R. gnavus* ATCC29149 (Owen et al., 2017) were all characterized as factors that could adhere to intestinal mucins. In fact, these above recombinant proteins bound to the mucus layer in murine colonic sections (Figure 1). Furthermore, Coïc et al. (2012) demonstrated that the modified MUB_70_ protein from *Lactobacillus reuteri* specifically binds to MUC2, the most abundant secreted mucin, and also reacts to mucosal section from patients diagnosed with colonic mucinous carcinoma. This study also highlighted MUB_70_ as a marker for mucinous carcinomas.

### Cell Lines and Intestinal Organoids

The human intestinal cell lines Caco-2 and HT-29, isolated from colon adenocarcinomas, are most widely used to investigate the adhesion of commensal and the invasion/adhesion of pathogens to intestinal epithelial cells (Parlesak et al., 2004; CenciČ and Langerholc, 2010). Caco-2 cells form polarized monolayers in culture and differentiate into enterocyte-like intestinal epithelial cells, while HT-29 cells in culture remain essentially undifferentiated (Rousset, 1986). Generally, the adhesion levels of bacteria are determined *via* viable cell counts, quantitative PCR, crystal violet staining, or mucin-binding assays. Additionally, the pathogenic invasion (such as *Salmonella*, *Campylobacter*, and enteropathogenic *E. coli*) of Caco-2 and HT-29 cells is determined using the gentamicin-protective assay (Steele-Mortimer, 2008). HT-29 cells secreting a small amount of mucus were selected based on their goblet cell-like phenotype using methotrexate (called MT-29 MTX). HT-29 MTX cells secrete the gastric mucin MUC5AC rather than MUC2, forming a 3 to 5μm thick mucus layer (Navabi et al., 2013); therefore, they are often used as a cellular model to study mucin secretion (Etienne-Mesmin et al., 2019).

The recently developed organoid technology, which allows the propagation of the colonic epithelium, enables the generation of self-propagating spheres of primary intestinal epithelial cells. Moreover, re-constructed monolayered organoids with their apical sides directly exposed to the culture medium can be used to evaluate the interactions between bacteria and the epithelial surface (Sato et al., 2011; In et al., 2016; Nakamoto et al., 2019). Recently, “IHACS,” a co-culture model of colonic organoids and anaerobic bacteria, was developed to ensure the maintenance of the apical side of organoids under an anaerobic state (Sasaki et al., 2020). Based on the conflicting oxygen demands between the epithelium and anaerobic bacteria, this model allows for the evaluation of anaerobic bacteria (such as *A. muciniphila*, *Bifidobacterium adolescentis*, *B. fragilis*, and *Clostridium butyricum*)-epithelial cell interactions under physiological conditions (Sasaki et al., 2020).

### Fecal Fermentation Systems

Several fecal fermentation systems that simulate the GI microbiota have been widely used to evaluate drug metabolism (Javdan et al., 2020) and the effect of exogenous functional compounds, such as prebiotics on the microbiome composition (Sasaki et al., 2018, 2019). Basically, glycerol-stocked human feces are suspended in bacterial culture medium and anaerobically cultured in DURAN^®^ bottles or jar fermenters. The human intestinal microbiota model “KUHIMM” is a single-batch fermentation system, which mimics the human colonic microbiota, allowing to reach densities of up to 10^11^ cells/ml in 24h (composed of more than 500 microbial species; Figure 1; Takagi et al., 2016). Interestingly, *Bifidobacterium* exhibited a distinct extracellular appendage in co-culture with fecal microorganisms versus *in vitro* mono-cultures. *Bifidobacterium longum* secreted several mucin adhesive proteins *via* EVs in the abovementioned system, but not in basal medium, suggesting that symbiotic bacteria within the microbiome induced this particular bacterial phenotype (Nishiyama et al., 2020). A mucosal simulator of the human intestinal microbial ecosystem “M-SHIME” has been developed (Van den Abbeele et al., 2012); an artificial system consisting of carrier material coated with commercial porcine gastric mucins. Bacteroidetes and Proteobacteria were enriched in the luminal compartment while Firmicutes colonized the mucin layer; interestingly, *Clostridium* cluster XIVa accounted for almost 60% of the M-SHIME mucus-adhered microbiota (Van den Abbeele et al., 2013). The previously described *in vitro* bacterial adhesion models mainly focus on bacteria-host interactions, while the fermentation systems allow the consideration of co-existing bacteria and the impact of the expected complexity on bacterial adhesion.

### Bacteria-Mimicking Microparticles

Microbeads of 1–10μm diameter can be modified with proteins and antibodies, conferring biological functionality to investigate the interactions with enterocytes and mucus. Generally, carboxy- or aldehyde-functionalized polystyrene microbeads are covalently coupled to proteins (Huebinger et al., 2016; Nishiyama et al., 2019; Kuhn et al., 2020). Polystyrene microbeads treated with borate buffer may also be coupled with protein *via* hydrophobic interactions (Oelke et al., 2003). For instance, microbeads modified with the multivalent adhesion molecule MAM7 reportedly prevented multidrug-resistant *Pseudomonas aeruginosa* infection in mice *via* their displacement from the host tissues (Huebinger et al., 2016). EVs from *Lactobacillus* coupled into microparticles showed anti-inflammatory effects, such as reducing the levels of pro-inflammatory TNF-α (versus native microparticles), highlighting these probiomimetics as strong candidates for translation (Kuhn et al., 2020).

Microbeads coupled with bacterial proteins (bacteria mimics) can also be used to determine their potential roles as adhesion factors *in vivo* and *in vitro*. The elongation factor Tu (EF-Tu), known as moonlighting adhesion factor, was coupled to microbeads, and the complex mimicked bacterial cell surface-localized protein. The modified microbeads adhered well to Caco-2 cells (compared with non-coated beads) *in vitro* and localized in the whole intestinal murine tissues *in vivo*, as per the fluorescence signals visualized with the optical clearing method (Nishiyama et al., 2019, 2020). Since this method allows to exclude external factors, such as stress resistance and bacterial survival, it enables the evaluation of the involvement of the cell surface architecture in bacterial adhesion.

### Limitations of *in vitro* Experimental Models Used to Study Host-Bacteria Interactions

*In vitro* experimental models are useful for understanding microbe-host interactions. Mucin-immobilized microtiter plates and cell lines are used to evaluate the adherence of commensal/pathogenic bacteria to mucosal surface *in vitro*. Meanwhile, these *in vitro* experimental models cannot completely represent the gut physiological conditions. For example, mammalian colonic mucus is primarily composed of Mucin-2 (MUC2; Johansson et al., 2008), which its *O*-glycosylation profile plays a critical role in the interaction between the bacteria and mucus (Donaldson et al., 2015; Jakobsson et al., 2015). The sulfation, sialylation, and fucosylation of *O*-glycans generate a diversity of MUC2 mucin (Bergstrom and Xia, 2013; Jin et al., 2017). However, significant glycosylation or mucosal site often occur between *in vitro* assays; therefore, caution is required when translating *in vitro* work into *in vivo*, due to limitations of individual method. Several experimental systems have been devolved to fill the gaps between traditional *in vitro* methods and gut ecological environments. For example, the fecal fermentation system allows for the co-existence of bacteria; the intestinal organoids can be used to assess details, such as health/disease states and mucin type (also glycosylation), for studying epithelial-bacterial responses; bacteria-mimicking microparticles can be used to evaluate the localization of specific adhesion factor from stomach to colon in mice. Therefore, to conduct research, the appropriate experimental method should be used.

## The Role of the Bacterial Cell Surface Architecture in Bacterial Colonization

Strategically localized cell surface architecture enables bacteria to be recognized by the host and promotes bacterial adhesion/colonization in the gut. Thus, the cell surface architecture plays an important role in microbe-host interactions. The involvement of adhesion factors in colonization has been well characterized in pathogenic bacteria. For example, Cholera is a foodborne infection that can be attributed to the ingestion of water or consumption of shellfish contaminated by *Vibrio cholerae* that possess two different adhesion factors, i.e., toxin-co-regulated pili (TCP pili) and *N*-acetylglucosamine-binding outer membrane protein (Klose, 2001; Kirn et al., 2005), promoting bacterial adhesion to the epithelial cells and mucus in the lower portion of the human GI tract. *V. cholerae* strains lacking these adhesions are defective in adherence to Caco-2 cells compared to the wild type. These mutants also hinder the colonization of the small intestine in the infant mouse model; they are expelled with the feces and are, thus, avirulent (Krebs and Taylor, 2011; Wong et al., 2012). Additionally, *Fusobacterium nucleatum*, a colorectal cancer-related bacterial species, adheres to the galactose-*N*-acetyl galactosamine structure expressed on the tumor surface *via* the outer membrane protein Fap2 (Coppenhagen-Glazer et al., 2015; Abed et al., 2016). *Fap2* gene-deficient *F. nucleatum* or Fap2 neutralization on the surface of wild-type *F. nucleatum via* the addition of galactose results in the reduced adhesion of *F. nucleatum* to tumor cells, promoting the decreased production of inflammatory cytokines thought to contribute to tumorigenesis (Casasanta et al., 2020). Collectively, these studies suggest that bacterial adhesion to the host mucus is a strategy used by pathogens during infection.

**Figure 2:**
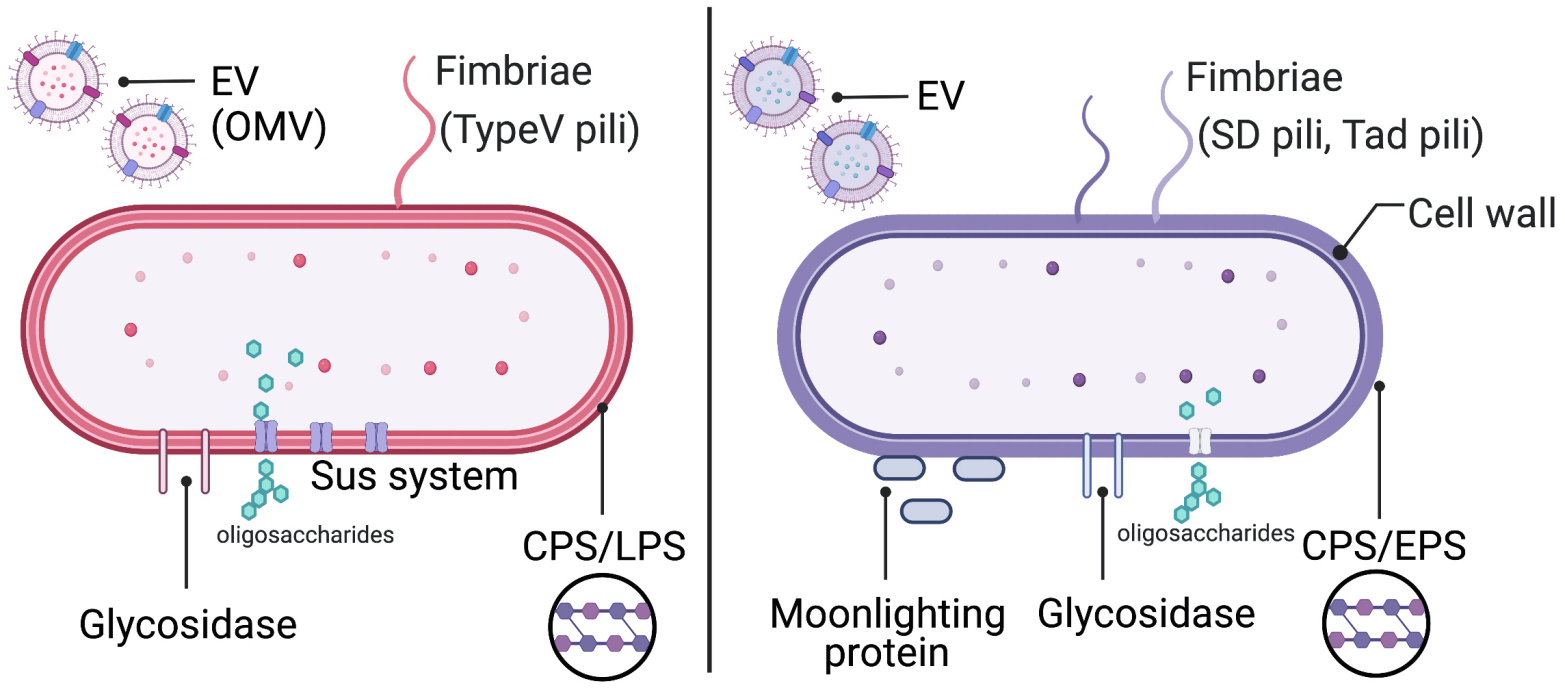
Cell surface architecture of *Bacteroides* (red) and *Bifidobacterium* (purple). EV, extracellular vesicle; Sus, starch utilization system; CPS, capsular polysaccharide; LPS, lipopolysaccharide; and EPS, exopolysaccharide. This figure was created using BioRender (https://app.biorender.com).

Here, we highlight the latest studies on the role of the bacterial cell surface architecture in colonization, especially in the mucus-associated *Bacteroides* and *Bifidobacterium* genera. The cell surface architectures of these bacterial species have been well characterized in the context of microbe-host interactions (Figure 2). The cell surface architectures of *Bacteroides* and *Bifidobacterium* is mainly classified into five types, namely, fimbriae/pili, glycosidases (including sugar transporters), multi-functional cytoplasmic proteins called “moonlighting factors,” and cell surface polysaccharides [capsular polysaccharides (CPS), lipopolysaccharides (LPS), and exopolysaccharides (EPS); Kline et al., 2009; Sonnenburg et al., 2010; Fagan and Fairweather, 2014; Donaldson et al., 2015; Nishiyama et al., 2016a; Etienne-Mesmin et al., 2019]. Outer Membrane Vesicles (OMVs)/EVs are also associated with the cell surface and, thus, may influence variations in cell surface architecture (Liu et al., 2018; Nishiyama et al., 2020; Palomino et al., 2021). Cell surface architecture differs based on the bacterial species, strains, or growth environment and is therefore a major factor in bacterial phenotype identification.

### Bacteroides

The phylum Bacteroidetes included members of the genus *Bacteroides*, typically the most abundant Gram-negative gut symbionts in the human microbiome. *Bacteroides* possess a dynamic cell surface architecture dependent on their CPS, LPS, and OMVs that promote interactions with the host but also mediate evasion from the host immune system (Cullen et al., 2015; Hickey et al., 2015; Porter et al., 2017). Of note, *B. thetaiotaomicron*, a predominant Bacteroidetes species, devotes ∼18% of its genes to the acquisition and utilization of a wide variety of carbohydrates (Martens et al., 2008), increasing the fitness of *B. thetaiotaomicron* in the GI tract (Xu et al., 2003; Martens et al., 2008; Foley et al., 2017; Schwalm and Groisman, 2017; Figure 2).

#### Capsular Polysaccharides/Lipopolysaccharide

*Bacteroides* species produce multiple CPS that play important roles during gut colonization (Krinos et al., 2001; Xu et al., 2003). The CPS structures appear to surround the entire cell (Tzianabossg et al., 1992), and the biosynthetic loci that encode these surface proteins are often under the control of phase variable promoters (Porter et al., 2017, 2020). A specific type of CPS (CPS5) is important in terms of adaptive immune responses as well as of antibiotic stress; CPS5-expressing *B. thetaiotaomicron* strain could increase anti-CPS IgA correlated with increased fitness in the mouse gut, thereby avoiding adaptive immunity. CPS5 also creates a mechanical barrier against antibiotic stress in several commensal *Bacteroides* (Porter et al., 2017). Additionally, the modification of LPS in Bacteroidetes also leads to resistance to inflammation-associated cationic antimicrobial peptides (AMP; Cullen et al., 2015); commensal Bacteroidetes express an enzyme, LpxF, that dephosphorylates the lipid A portion of LPS, leading to a decreased overall negative charge on the cell surface and increased resistance to cationic AMP, which was confirmed by *B. thetaiotaomicron lpxF* mutant strain (Cullen et al., 2015). These studies highlight mechanisms that ensure the stability of prominent commensal bacteria in the gut.

#### Outer Membrane Vesicles

Several commensal and pathogenic bacteria release extracellular particles, referred to as OMVs (only in Gram-negative bacteria), or EVs (Witwer and Théry, 2019), ranging from 20 to 400nm in diameter (Toyofuku et al., 2019). Among *Bacteroides* species, OMVs have been implicated in microbial and immune homeostasis. For instance, the CPS expressed on the surface of OMVs from *B. fragilis* was shown to promote both Treg cell responses and the production of interleukin (IL)-10 by dendritic cells (DC) through a toll like receptor 2-mediated mechanism, contributing to protection in a mouse model of acute colitis (Shen et al., 2012). This study revealed that *B. fragilis* packages CPS on OMVs potentially transport mechanism to deliver immunomodulatory signals to host cells. The purified OMVs from *B. thetaiotaomicron* also induced IL-10 expression and a regulatory phenotype in the DC of colonic biopsies from healthy subjects but not in those of patients with inflammatory bowel disease (Durant et al., 2020). The OMVs of *B. thetaiotaomicron* secrete several enzymes that can alter host physiology. *B. thetaiotaomicron* OMVs exhibited sulfatase activity, mediating bacteria-host immune cell interaction in a sulfatase-dependent manner and leading to the development of colitis in genetically susceptible *dnKO* mice (Hickey et al., 2015). Additionally, *B. thetaiotaomicron* was shown to secrete a cell-signaling InsP6 phosphatase (BtMinpp) *via* OMVs, which promotes Ca2^+^ signaling in intestinal epithelial cells; importantly, BtMinpp was packaged inside OMVs and thus, protected from degradation by host proteases (Stentz et al., 2014). These studies highlight OMVs and major players in the context of bacteria-host interactions; however, further studies are still needed to identify the various yet unknown molecules packaged into OMVs.

#### Glycosidases

The gut colonization ability of bacteria is also determined by their ability to utilize carbohydrates. The polysaccharide utilization loci (PULs) encode the enzyme and protein ensembles required for the saccharification of mucin glycoproteins (Lynch and Sonnenburg, 2012). *B. thetaiotaomicron* dedicates nearly a fifth of its 6.26 Mbp genome (∼18%) to 88 distinct PULs (Martens et al., 2008). Mechanistically, mucin is first degraded by extracellular glycosidases, such as PUL-encoded sulfatase and then internalized *via* the starch utilization system (Sus) transporter, consisting of the substrate-binding factor SusD and the transmembrane component SusC. Finally, polysaccharides are degraded into mono- or disaccharides used as a carbon source by *Bacteroides*. These metabolic processes are under the control of a two-component sensor regulator (TCS); in the presence of “high-priority nutrients,” such as chondroitin sulfate and heparin glycosaminoglycans, the expression of PUL is strongly activated (Sonnenburg et al., 2010; Pudlo et al., 2015). Mucin-derived free-sialic acid and fucose, which was liberated by *Bacteroides*, are not only used as the carbon source by *Bacteroides* but also by pathogenic bacteria, such as *Salmonella* Typhimurium and *Clostridioides difficile via* cross-feeding in the mouse gut (Ng et al., 2013).

Bacterial species-specific carbohydrate utilization systems known as commensal colonization factors (CCFs) have been identified in *B. fragilis* and *B. vulgatus*. CCFs enable these bacteria to colonize saturable nutrient niches (Lee et al., 2013; Donaldson et al., 2018). Deletion of *ccf* in *B. fragilis* caused colonization defects in mice and reduced horizontal transmission. Similarly, the CCF system was also required for the penetration of *B. fragilis* into the mice colonic crypts and for long-term resilience to intestinal perturbations, such as antibiotic treatment, suggesting that the niche within the colonic crypts represents a reservoir of bacteria, essential for the maintenance of long-term and stable colonization (Lee et al., 2013).

**Figure 3:**
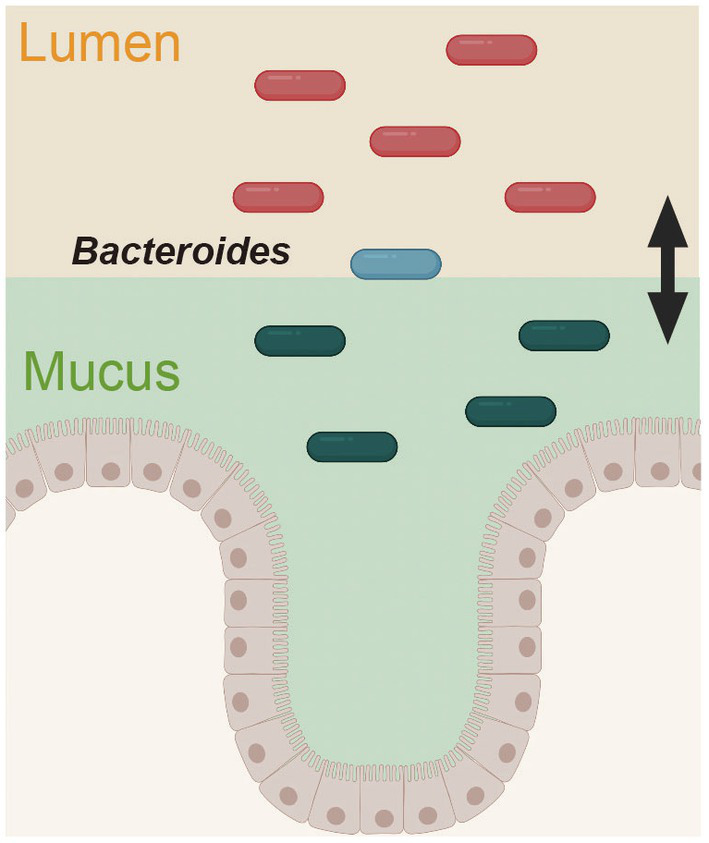
Spatially distinct physiology of *Bacteroides fragilis* during colonization. The gene expression pattern in *B. fragilis* was distinct in the mucus layer and epithelium versus the intestinal lumen. This figure was adapted from Donaldson et al. (2020) and created using BioRender (https://app.biorender.com).

Recently, two glycolytic enzymes from *B. fragilis* were further identified as colonization factors (Donaldson et al., 2020). The authors of this study focused on the differences in bacterial communities across the gut. The intestinal lumen, mucus layer, and epithelial cells of *B. fragilis*-administered mice were analyzed by hybrid selection RNA sequencing (hsRNA-Seq). Thus, the transcript-level expression of sulfatase (BF3086) and glycosyl hydrolase (BF3143) was remarkably increased in the mucin layer and epithelium compared with the intestinal lumen; interestingly, the expression of these genes did not change in the intestinal lumen but increased in the mucin layer and epithelial cells. The deletion of these genes impaired mucosal colonization *in vivo*, thus revealing a new site-specific gene expression determinant of bacterial colonization. This adaptive mechanism of *B. fragilis* in response to the environment provides a unique perspective for understanding bacterial colonization in the intestine (Figure 3).

#### Fimbriae

Many Gram-negative and -positive bacteria (also refer to the following section) species possess fimbrial rod structures known as fimbriae extending from their surfaces. Therefore, it is not surprising that fimbriae (pili) are among the most characterized adhesion factors in commensal and pathogenic bacteria. Adhesive fimbriae can be placed into four distinct groups based on their assembly pathways, namely, chaperone-usher pili (Type I pili), Type IV pili, curli pili, and Type V pili in Gram-negative bacteria (Fronzes et al., 2008). Type I pili are assembled *via* the chaperone-usher pathway, and the most characterized adhesive pili in Enterobacteriaceae, such as *Salmonella* and *Escherichia* spp.; they consist of hundreds to thousands of subunits forming a single hair-like pilus that is closely related to virulence (Proft and Baker, 2008; Kline et al., 2009). Type V pili were recently found in the Bacteroidetes class, especially in *Porphyromonas gingivalis* (Xu et al., 2016); they assemble *via* protease-mediated polymerization and are also a key determinant of *P. gingivalis* virulence (Shibata et al., 2020). Type V pili-related genes were also discovered in the genome of gut commensal bacteria, such as *Bacteroides* and *Prevotella* (Shoji et al., 2020). Using transposon mutagenesis followed by a biofilm positive-selection procedure using microtiter-plate biofilm assay and crystal violet staining, Mihajlovic et al. (2019) discovered that Type V pili potentially contribute to *B. thetaiotaomicron* biofilm formation and adhesion capacity. However, the role of Type V pili with regard to *Bacteroides* colonization is mostly unknown; further studies are needed, beginning by the detailed functional characterization of *Bacteroides* Type V pili.

### Bifidobacterium

Within the phylum Actinobacteria, members of the genus *Bifidobacterium* are particularly abundant in the infant’s gut as Gram-positive symbionts. *Bifidobacterium* expresses a variety of extracellular and cytoplasmic glycosyl hydrolases (GHs) important for the hydrolysis of oligosaccharides within mucin glycans as well as of human milk oligosaccharides (HMOs) for energy generation (O’Callaghan and van Sinderen, 2016). Extracellular GHs are mainly found in the *B. bifidum* and not in other *Bifidobacterium* species, such as *B. longum*, *Bifidobacterium infantis*, and *Bifidobacterium breve* (Kiyohara et al., 2012; Gotoh et al., 2019). In addition to the GHs, *Bifidobacterium* possesses several cell surface components, including CPS/EPS (Fanning et al., 2012; Tahoun et al., 2017) and fimbriae (O’Connell Motherway et al., 2011; Turroni et al., 2013), which are important for the fitness of *Bifidobacterium* strains in the GI tract. Moonlighting factors and the production of EVs were also reported as an assist bifidobacterial colonization in *Bifidobacterium* species (Nishiyama et al., 2020; Figure 2).

#### Capsular Polysaccharides/Exopolysaccharides

*Bifidobacterium* spp.-derived EPS plays a role in the host-microbe interactions essential for bacterial adhesion and the consequent colonization of the GI tract; additionally, they also play immunomodulatory roles (Fanning et al., 2012; Tahoun et al., 2017; Verma et al., 2018). The *B. breve* strain UCC2003 produces EPS, which provides stress tolerance as well as acid and bile resistance (Fanning et al., 2012). In addition, this strain can evade B cell responses and consequently colonize the mouse GI tract for the long-term, which is not the case for EPS-deficient strains (Fanning et al., 2012). *B. longum* 105-A produces CPS and EPS that negatively correlated with bacteria-host interactions *in vitro*. Wild-type *B. longum* 105-A could not adhere to Caco-2 cells, while CPS mutant strains do, *via* fimbriae formation (Tahoun et al., 2017). These studies suggest that cell surface EPS and CPS impact the persistence of *Bifidobacterium* in the gut, but not the initial adhesion. Based on the structural analysis of polysaccharides, *B. bifidum* PRI1 produces two types of complex mixtures of polysaccharides, namely, phospho-glycero-β-galactofuranan (PGβG) and one composed of four neutral polysaccharides (CSGG); these mixtures can differently induce the immune response (Speciale et al., 2019). PGβG enhanced pro-inflammatory immune responses by increasing interferon-γ levels, while CSGG efficiently induces Treg cells through a partially TLR2-mediated mechanism by ameliorating intestinal inflammation (Verma et al., 2018; Speciale et al., 2019). These polysaccharide compositions have been analyzed using culture medium, with possibly different *in vivo* and *in vitro* composition. In the intestinal tract, polysaccharides are heavily influenced by diet and symbiotic microorganisms.

#### Glycosidases

Among *Bifidobacterium* species, *B. bifidum* expresses several extracellular glycosidases to degrade host-derived glycans, including HMOs, mucin *O*-glycans, and glycosphingolipids (Gotoh et al., 2019; Katoh et al., 2020). Cross-feeding of the oligosaccharide degradants among bifidobacterial communities has, in fact, been characterized. For example, galacto-*N*-biose (GNB) is a core 1 disaccharide of *O*-glycans within mucin glycoproteins present in the human colon and breast milk (Podolsky, 1985). Lacto-*N*-biose (LNB) is also abundant in breast milk, especially the colostrum (Erney et al., 2000). Remarkably, LNB and GNB disaccharides liberated *via* the action of lacto-*N*-biosidases (Wada et al., 2008) or endo-α-*N*-acetylgalactosaminidases (Fujita et al., 2005) from *B. bifidum* are important for the cross-feeding of *B. longum*, as confirmed using the GNB/LNB transporter-deficient *B. longum ΔgltA* strain *in vitro* culture (Katoh et al., 2020). In addition, mucin-related sialic acid liberated by *B. bifidum via* extracellular sialidases was shown to promote the growth of *B. breve* (also *via* cross-feeding; Egan et al., 2014; Nishiyama et al., 2018), which cannot grow in the presence of mucin as the sole carbon source *in vitro* culture. These observations suggest that the extracellular glycosidases of *B. bifidum* may help elucidate the complex mechanism by which *Bifidobacterium* communities cross-feed and develop in the gut (Gotoh et al., 2019).

The extracellular enzyme sialidase (SiaBb2) of *B. bifidum*, rather than degrading intestinal carbohydrates, acts as a lectin-like adhesion factor capable of binding to sialylated and blood group sugar chain, as confirmed using glycan array with recombinant SiaBb2 protein (Nishiyama et al., 2017). The *R. gnavus trans*-sialidase RgNanH was also characterized as an adhesion factor toward sialic acid-rich mucus; the CBM40 region of RgNanH binds to mouse mucosal tissue section and to purified mucins from LS174T cell line. CBM40 specifically binds to α2-3-sialylated Lewis X, which prefers Neu5Ac than Neu5Gc (Owen et al., 2017). Extracellular sialidases are found in several commensals (El Kaoutari et al., 2013; Juge et al., 2016); therefore, they probably have a transversal role in the adhesion-mediated bacterial colonization of the gut.

#### Fimbriae

Gram-positive bacteria belonging to the Actinomyces and Firmicutes phyla, such as *Actinomyces*, *Bifidobacterium*, *Corynebacterium*, *Enterococcus*, *Lactobacillus*, and *Streptococcus*, possess sortase-dependent fimbriae/pili (SD pili; Hendrickx et al., 2011; Khare and Narayana, 2017). Type IV pili were also identified in several Gram-positive species, such as *Clostridium perfringens* (Varga et al., 2006) and *C. difficile* (McKee et al., 2018). *Bifidobacterium* possesses two types of fimbriae: SD pili and Type IV pili (Tad pili; O’Connell Motherway et al., 2011; Turroni et al., 2013; Milani et al., 2017). For instance, the SD pili (Fim) from *B. bifidum* adheres to human epithelial cell lines and extracellular matrix proteins especially fibronectin, contributing to bacterial aggregation (Turroni et al., 2013). The author also indicated that since deglycosylation of fibronectin caused a reduction in the *Lactococcus lactis* heterologous *fim* expression strain, Fim protein might recognize the carbohydrate residues of fibronectin (Turroni et al., 2013). The FimA subunit of *B. longum* SD pili can be classified into five types based on polymorphisms on the amino acid sequence (Type-A-E; Iguchi et al., 2011). Based on the BIAcore assay, type-A FimA recombinant protein showed a dissociation constant as low as 10^8^M^−1^ to porcine colonic mucin, while type-B and -C FimA bound weakly to the same mucin moiety (Suzuki et al., 2015), suggesting that FimA is a lectin-like protein that strictly recognizes host carbohydrates chains. The expression of SD pili in the presence of lysine (Turroni et al., 2013) or xylan (Milani et al., 2017) was confirmed *via* atomic force microscopy. In addition, *B. breve* use Tad pili to colonize and persist in the murine intestinal tract (O’Connell Motherway et al., 2011); interesting enough, Tad pili also contribute to the maturation of the naïve gut (neonatal mucosa) *via* the stimulation of colonic epithelial proliferation, as per the results of *in vivo* and *in vitro* experiments (O’Connell Motherway et al., 2019).

#### Moonlighting Factors

Moonlighting factors are proteins that exhibit different (or multi) functions other than their main ones and are closely related to various biological activities in bacteria, yeast, and plants (Jeffery, 1999; Huberts and van der Klei, 2010; Kainulainen and Korhonen, 2014). Most of the moonlighting factors in bacteria are cytoplasmic proteins that have essential functions for bacterial growth, such as metabolic enzymes, molecular chaperones, and transcription factors. For instance, several cytoplasmic proteins exert additional functions, including the promotion of bacterial adhesion to the host, with a potential impact on gut colonization. This hypothesis was well explored in *Bifidobacterium*. For instance, the transaldolase from *B. bifidum* A8, when secreted to the extracellular milieu promotes the adhesion and aggregation of bacteria to mucin, which was confirmed using mucin-immobilized microtiter plates (González-Rodríguez et al., 2012). In addition, the DnaK, and enolase from *Bifidobacterium animalis* subsp. *lactis* BI07, binds to surface-exposed human plasminogen (Candela et al., 2009, 2010). The author also showed that positively charged residues as well as the negatively charged-specific amino acids of *B. lactis* BI07 enolase are essential for plasminogen binding (Candela et al., 2009). These proteins are involved in glycolysis, energy generation, and protein folding, suggesting that *Bifidobacterium* may increase the functional range of a limited set of proteins (moonlighting factors) to ensure survival within the GI tract.

#### Extracellular Vesicles

Membrane budding and the release of spherical particles into the surrounding extracellular milieu were observed in several species of Gram-positive bacteria (Firmicutes and Actinobacteria phyla; Liu et al., 2018). Importantly, several studies have reported the therapeutic applications of bifidobacterial EVs. For instance, *B. bifidum* LMG13195-derived EVs induce Treg polarization, suggesting these EVs may be useful adjuvants for immunotherapy (López et al., 2012). *B. longum* KACC 91563-derived EVs containing a family of five solute-binding proteins reduced the occurrence of diarrhea in a food allergy murine model (Kim et al., 2016). Similar immunomodulatory effects were also reported for EVs derived from several probiotic bacteria, such as *L. rhamnosus* – EVs enriched in 60 heat-shock proteins probably responsible for the observed phenotypic change in dendritic cell co-cultures (Al-Nedawi et al., 2015). EVs released from *Lactobacillus sakei* were associated with an increase in IgA production *via* the activation of TLR2 signaling (Yamasaki-Yashiki et al., 2019). Altogether, these studies suggest that EVs act as carriers of various cytoplasmic or cell surface components, with potential functions that promote bacterial persistence. Notably, the secretion of EVs by *Bifidobacterium* is a documented phenomenon; however, the ecological impact is still uncertain as the factors responsible for EV production have yet to be identified.

**Figure 4:**
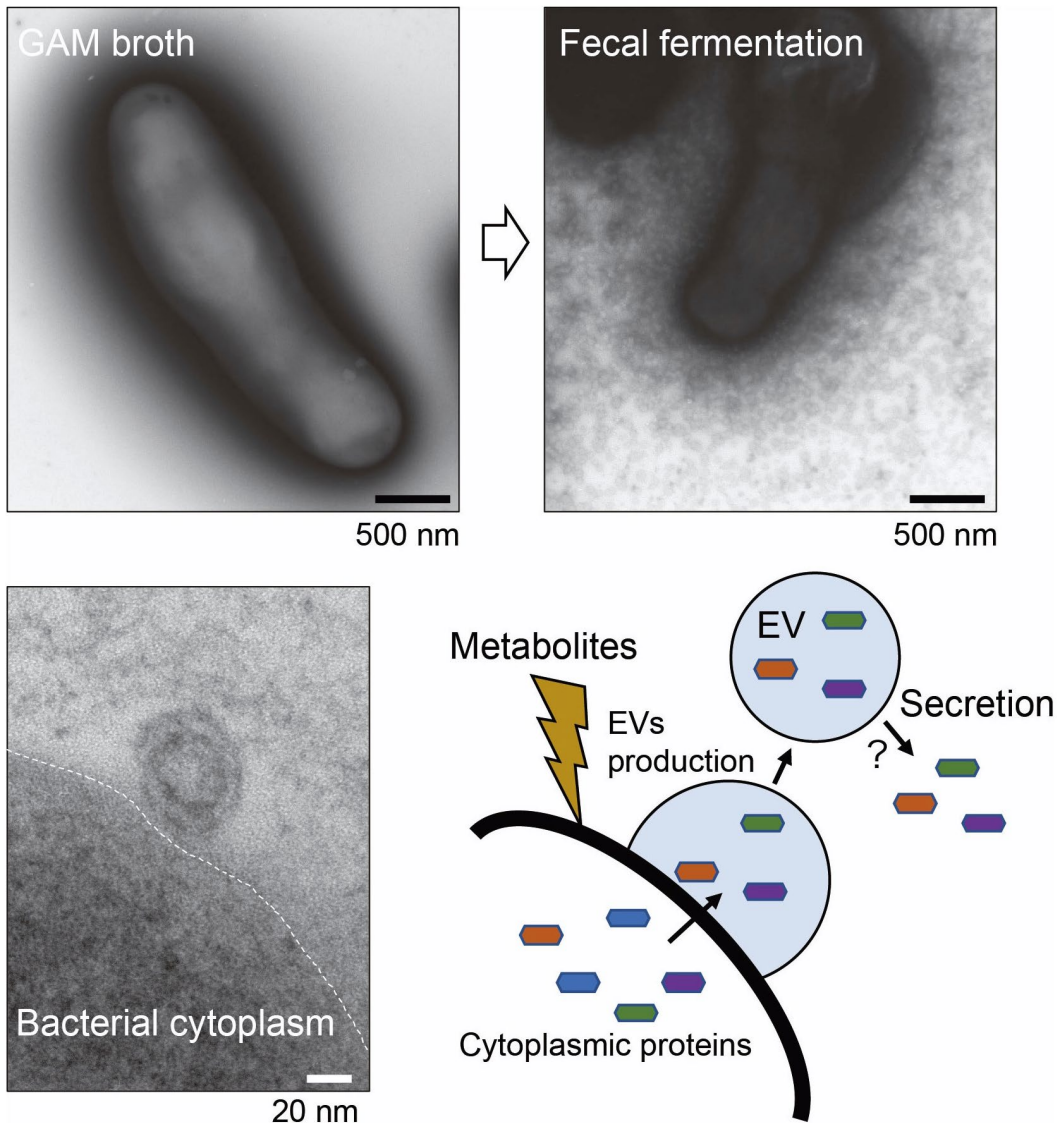
Secretion of extracellular vesicles (EVs) by *Bifidobacterium longum*. Upper panels: *B. longum* NC2705 whole cells were negatively stained with uranyl acetate and examined under a TEM. Lower panel: ultrathin section of *B. longum* examined under a TEM. *B. longum* cultured in bacteria-free fecal fermentation broth secreted a myriad of EVs, probably in response to symbiotic microbiota-derived metabolites. The EVs contain several adhesive moonlighting proteins that may promote bifidobacterial colonization. This figure was adapted from Nishiyama et al. (2020) and Copyright © 2020 Kagakutoseibutsu (DOI: 10.1271/kagakutoseibutsu.58.614) with several modifications.

Recently, electron micrograph of *B. longum* NCC2705 whole cells with negative staining revealed that *B. longum* released a myriad of EVs when cultured in a bacteria-free fecal fermentation broth (Figure 4). The *B. longum* EVs production pattern differed among individual human fecal samples suggesting that metabolites derived from symbiotic microbiota stimulate the active production of EVs (Nishiyama et al., 2020). EVs were also collected using ultracentrifugation from *B. longum* NCC2705 suspension, while the protein composition of EVs was determined using the combination method of proteomics and BIAcore analyses (BIA-MS). Importantly, the results of BIA-MS showed that the EVs primarily consisted of cytoplasmic proteins, including several mucin-binding proteins, such as phosphoketolase, GroEL, EF-Tu, phosphoglycerate kinase, transaldolase, and heat-shock protein 20. This result suggesting that the EVs-mediated exportation of adhesive moonlighting proteins may assist bifidobacterial colonization (Figure 4). However, the secretory mechanism of these moonlighting cytoplasmic proteins from EVs is mostly unknown. Therefore, further studies are needed to elucidate the abovementioned mechanism; then, we might be able to deeply understand the role of EVs and moonlighting proteins in the ecological niche.

## Adhesion of *Bacteroides* and *Bifidobacterium* to Dietary Glycans

Symbiotic mucosal bacteria can also use nondigestible carbohydrates, including those in dietary fiber from plants, bacteria, and fungi, as well as in animal-derived cartilage and tissues and chemically synthesized carbohydrates. These include plant-derived non-starch polysaccharides, such as cellulose, hemicellulose (the two with a high abundance of polysaccharides), inulin, gum, and pectin, which display a substantial variety of chemical structures (Raigond et al., 2015; Deehan et al., 2017). Importantly, glycan-mediated bacterial adhesion is one of the primary means by which bacteria increase their access to nutrients (Martens et al., 2014; Rakoff-Nahoum et al., 2014; Patnode et al., 2019).

*Bacteroides* PULs are important for the breakdown of a variety of plant as well as intestinal mucin glycans (Koropatkin et al., 2012; Wexler and Goodman, 2017). For instance, the outer membrane lipoproteins (SusD, SusE, and SusF) encoded by PULs are involved in the initial binding to starch polysaccharides (Anderson and Salyers, 1989). These Sus-like systems are widespread among the Bacteroidetes members in the human gut and are unique to this phylum (Xu et al., 2007; Martens et al., 2009, 2011). Sus with substrate specificities for plant cell wall polysaccharides, such as arabinan, xylan, β-glucan, and glucomannan, has been identified in Bacteroidetes (Martens et al., 2011). Interestingly, *B. thetaiotaomicron* can also metabolize and use α-mannan derived from co-existing the yeast *Saccharomyces cerevisiae via* Sus enzymatic redundant degradation steps (Cuskin et al., 2015). Recently, Patnode and colleagues revealed that 160 *Bacteroides* and *Parabacteroides* strains show diverse strain- and glycan-specific binding phenotypes using glycan arrays and glycan-immobilized beads; specific bacterial strains bound to distinct carbohydrate structures, explaining the strain-specific differences in the catabolism of glycans and adhesion (Patnode et al., 2021).

*Bifidobacterium* were also reported to adhere to dietary glycans, such as cellulose and chitin (Taniguchi et al., 2021). However, in contrast to *Bacteroides*, which possess sophisticated PULs to degrade and import dietary polysaccharides (irrespectively of their origin and type), *Bifidobacterium* uses only specific polysaccharide-degradation pathways which vary depending on the strain/species. For instance, *B. breve* UCC2003 possess an extracellular bifunctional amylopullulanase (ApuB), composed of a distinct α-amylase-containing domain that hydrolyzes starch (and related polysaccharides) and a C-terminally located pullulanase-containing domain, which hydrolyzes pullulan. ApuB can bind to and degrade starch, amylopectin, glycogen, or pullulan, thus promoting the assimilation of dietary glycans by *B. breve* (O’Connell Motherway et al., 2008). These α-amylase and/or pullulanase activities were mostly found in *B. breve* (and not in other *Bifidobacterium* strains; Ryan et al., 2006). Gum arabic arabinogalactan (AG) is a widely distributed plant fiber. Curiously, extracellular 3-*O*-α-D-galactosyl-α-L-arabinofuranosidase (GAfase) is involved in the assimilation of AG in *B. longum* JCM 7052, while *B. longum* JCM 1217 (lacking GAfase) cannot assimilate AG (Sasaki et al., 2021). In line with this, the distribution of GAfase encoding homologous genes was conserved in only 6.84% of *B. longum* strains (*n*=307) as per the NCBI database (Sasaki et al., 2021). This fact suggests a survival strategy of bifidobacteria based on the selection of the genes necessary for the adaptation to the surrounding environment (Odamaki et al., 2018).

## Conclusion and Outlook

In this manuscript, we focused on the colonization properties of mucosal bacteria, particularly on the roles of the cell surface architecture, with a particular focus on *Bacteroides* and *Bifidobacterium*. Bacterial adhesion to host glycans or dietary fiber is seemingly simple; various cell surface components are essential for interaction-mediated adhesion. Importantly, the physiological properties of the cell surface architecture components dramatically change depending on the co-existing symbiotic bacteria and of the spatial distribution within the mucosal niche. Therefore, these facts clearly suggest that bacteria exhibit a remarkable adaptability, allowing their establishment in different environments and the maintenance of symbiotic relationships with other bacteria and the host. The analysis of such symbiotic relationships is, therefore, essential for a comprehensive understanding of the intestinal ecosystem. To clarify such interactions, several devices/methods have been developed, such as the KUHIMM culture system that imitates the bacterial flora in the human large intestine (Takagi et al., 2016), and the co-culture of anaerobic bacteria and organoids (Sasaki et al., 2020). New genetic engineering technologies also applicable to bacteria (including anaerobes) have been successively established (Mimee et al., 2015; Lim et al., 2017; Guo et al., 2019). Therefore, we shall take advantage of the newly developed technologies to completely characterize the bacterial cell surface architecture, aiming to fully understand the bacteria-bacteria and bacteria-host symbiotic interactions. We believe that the adoption of a cross-sectional approach based on phenotypes is of paramount importance.

## Author Contributions

KN, TY, MS, RO, TM, and NO provided ideas and contributed to the structure of this manuscript. KN, TY, and MS wrote the manuscript. RO, TM, and NO reviewed the manuscript before submission for its intellectual content. All authors contributed to the article and approved the submitted version.

## Funding

This work was supported by a Grant-in-Aid for Young Scientists (20K15438) from the Japan Society for the Promotion of Science. This work was also partially supported by the Institute for Fermentation, Osaka (IFO).

## Conflict of Interest

The authors declare that the research was conducted in the absence of any commercial or financial relationships that could be construed as a potential conflict of interest.

## Publisher’s Note

All claims expressed in this article are solely those of the authors and do not necessarily represent those of their affiliated organizations, or those of the publisher, the editors and the reviewers. Any product that may be evaluated in this article, or claim that may be made by its manufacturer, is not guaranteed or endorsed by the publisher.
